# A drug free solution for improving the quality of life of fibromyalgia patients (Fibrepik): study protocol of a multicenter, randomized, controlled effectiveness trial

**DOI:** 10.1186/s13063-022-06693-z

**Published:** 2022-09-05

**Authors:** Emilie Chipon, Jean-Luc Bosson, Laure Minier, Anne Dumolard, Antoine Vilotitch, David Crouzier, Caroline Maindet

**Affiliations:** 1 REMEDEE LABS SA, Montbonnot-Saint-Martin, France; 2 TIMC Laboratory CNRS-UMR 5525, University Grenoble Alpes, Grenoble, France; 3 Public Health Department, CHU Grenoble Alpes, 38000 Grenoble, France; 4 Pain Medicine Department, CHU Grenoble Alpes, 38000 Grenoble, France; 5 Data engineering cell - CHU Grenoble Alpes, 38000 Grenoble, France

**Keywords:** Fibromyalgia, Quality of life, Millimeter waves, Endorphins, Pain, Sleep disturbances, Holistic approach

## Abstract

**Background:**

Fibromyalgia is a form of chronic widespread pain that is defined as a syndrome of chronic symptoms of moderate to severe intensity, including diffuse pain, fatigue, sleep disturbance, cognitive impairment, and numerous somatic complaints. To date, there is no specific drug treatment for fibromyalgia but only symptomatic treatments. A drug free solution based on a wristband that emits millimeter waves associated with a therapeutic coaching program was developed. The application of millimeter waves on an innervated area has been described to have a neuromodulating effect, due to endorphin release stimulation and parasympathetic activation. Coaching is carried out to improve the patient’s adherence and to increase compliance and effectiveness of the treatment. Regular use of this solution by fibromyalgia patients is expected to improve sleep quality, reduce anxiety and pain levels, and, at the end, increase the quality of life.

**Methods:**

This trial is performed over 8 French inclusion centers for a total of 170 patients. The effectiveness of the solution is evaluated according to the primary objective, the improvement of the quality of life measured through the dedicated Fibromyalgia Impact Questionnaire after 3 months. Patients are randomized in two groups, Immediate or Delayed. The Immediate group has access to the solution just after randomization in addition to standard care, while Delayed has access to the standard of care and waits for 3 months to have the solution. The purpose of this methodology is to limit deception bias and facilitate inclusion. The solution consists in using the device for three sessions of 30 min per day and four coaching sessions spread over the first 2 months of wristband usage.

**Discussion:**

The objective is to confirm the effect of the integrative approach based on endorphin stimulation and a therapeutic coaching program in nociplastic pain and specifically for the patient suffering from fibromyalgia. If the effectiveness of the solution is demonstrated, we will be able to respond to the demand of fibromyalgia patients for access to an effective non-medicinal treatment to improve their quality of life.

**Trial registration:**

ClinicalTrials.gov NCT05058092

**Supplementary Information:**

The online version contains supplementary material available at 10.1186/s13063-022-06693-z.

## Administrative information

Note: the numbers in curly brackets in this protocol refer to SPIRIT checklist item numbers. The order of the items has been modified to group similar items (see http://www.equator-network.org/reporting-guidelines/spirit-2013-statement-defining-standard-protocol-items-for-clinical-trials/).

**Table:** 

Title {1}	A drug free solution for improving the quality of life of fibromyalgia patients (Fibrepik): study protocol of a multicenter, randomized, controlled effectiveness trial.
Trial registration {2a and 2b}.	ClinicalTrials.gov NCT05058092
Protocol version {3}	V3.0 - March 11, 2022
Funding {4}	Sponsor's own funds
Author details {5a}	CHIPON Emilie^1^, BOSSON Jean-Luc^2^, MINIER Laure^1^, DUMOLARD Anne^3^, VILOTITCH Antoine^4^, CROUZIER David^1^, MAINDET Caroline^5^ ^1^ REMEDEE LABS SA, Montbonnot-Saint Martin, France; ^2^ TIMC Laboratory CNRS-UMR 5525, Univ. Grenoble Alpes; Public Health Department, CHU Grenoble Alpes, Grenoble, 38000 France; ^3^ Pain Medicine Department, CHU Grenoble Alpes, Grenoble, 38000 France; ^4^ Data engineering cell - CHU Grenoble Alpes, Grenoble, 38000 France; ^5^ Pain Medicine Department, CHU Grenoble Alpes, Grenoble, 38000 France; TIMC Laboratory CNRS-UMR 5525, Univ. Grenoble Alpes.
Name and contact information for the trial sponsor {5b}	CHIPON Emilie – emilie.chipon@remedee.com
Role of sponsor {5c}	The sponsor participated in the meetings to set up the methodology, but it is not involved in the collection, analysis, and interpretation of data.

## Introduction

### Background and rationale {6a}

Fibromyalgia (FM) is a chronic rheumatic condition with an estimated prevalence between 2 and 6,4% depending on the criteria used in diagnosis [1]. The main symptoms of FM are widespread musculoskeletal pain, stiffness, fatigue, non-restorative sleep, mood disorders, cognitive dysfunction, anxiety, depression, general sensitivity, and physical and emotional inability to carry out normal daily activities [2–4]. Because fibromyalgia is a multifaceted disease in which the pathogenesis is still unclear, no dedicated curative treatment for this disease has been validated. As for most of the nociplastic pain, the main therapeutical strategy is focused on symptom mitigation and quality of life (QoL) improvement. In 2017, the European Alliance of Associations for Rheumatology (EULAR) updated its recommendations for the management of FM based on scientific evidence from high-quality reviews and meta-analyses [5]. In first-line, the EULAR recommended to inform patients by providing therapeutic education and then prescribing non-pharmacological therapies such as aerobic and strengthening exercise that can be combined with other non-drug therapies like cognitive behavioral therapies, acupuncture, hydrotherapy, massage, meditative movement therapies, and mindfulness-based stress reduction [5]. Drug treatments such as opioid analgesics, antidepressants, and anti-epileptics are used as second-line treatments proposed by the EULAR, as they do not always give convincing results and present significant risks for patients. Their prescription is recommended only when patients are in severe pain (duloxetine, pregabalin, tramadol) and/or in case of sleep disturbances (amitriptyline, cyclobenzaprine, pregabalin) [5].

The non-drug therapeutic solution proposed is totally in line with the proposals made by EULAR. It is based on a solution that includes a wristband, a mobile application, and a therapeutic coaching program. The wristband emits millimeter waves (MMW) that stimulate the nerve endings located in the skin [6]. This stimulation induces a series of coordinated physiological actions, which in turn induce the synthesis and release of endogenous opioids (endorphins) that have a hypoalgesic effect [7, 8]. The effectiveness of MMW therapy on different types of pain has been demonstrated in several animal [9–11] and human studies [12–16]. Pre-clinical studies have shown that endogenous opioids play a role in the balance of sympathetic/parasympathetic activities, towards an inhibition of the sympathetic system and an activation of the parasympathetic system [17–20] and thereby participate in the modulation and regulation of stress [21]. The increase in parasympathetic activity also facilitates sleep onset and improves the quality of sleep [22]. Pain and sleep disorders are central symptoms in FM and are intimately linked. Indeed, experimental studies in humans and animals show that there is a relationship between disorders of the sleep-wake cycle and diffuse musculoskeletal pain [23]. A poor night’s sleep is often followed by an increase in pain intensity the next day. Inversely, a particularly painful day is often followed by a bad night [24]. In healthy subjects, sleep deprivation causes hyperalgesic changes [23]. Healthy subjects report symptoms like those reported by people suffering from fibromyalgia (musculoskeletal pain, fatigue, and mood disorders). These symptoms have a strong impact on the quality of life of fibromyalgia patients. Also, the parasympathetic effects of using the wristband would improve the patients' sleep, consequently their pain and overall quality of life.

However, chronic pain could not be addressed by taking into account the only physiological dimension. Chronic pain is a multi-dimension situation, physiological, psychological, and social [25]. It is why most advance clinical research in that field insists on the need to consider each of its dimensions.

In addition to the endorphin stimulation, the coaching program has several objectives. First, it provides therapeutic education about the wristband (mechanism of action, expected effects, technical aspects) to improve adherence and reduce apprehension and side effects. For this purpose, two sessions of coaching are proposed: when the wristband is delivered and after 7 days of start of use. The second objective is to improve compliance and effectiveness. Indeed, a meta-analysis evaluating the effects of ecological interventions (EMI: Ecological Momentary Intervention) based on the use of technological tools (websites, apps) on mental health (stress, anxiety, and depression) shows that the results can be up to 62% greater when the intervention involves the punctual assistance of a health professional compared to an intervention without a health professional [26]. The authors of the study suggest that this assistance could increase users’ motivation and adherence, which in turn could increase the effectiveness of the intervention. To this end, two follow-up phone calls are proposed: one after a month of usage and one after 2 months.

### Objectives {7}

Pre-clinical and clinical data show the hypoalgesic and parasympathetic effects of MMW exposure on healthy participants and patients with pain. By allowing the release of endogenous opioids, the MMW transmitter system could improve the quality of life of fibromyalgia patients, measurable by a decrease in the Fibromyalgia Impact Questionnaire (FIQ) score, and reduce their sleep disorders, observable by a decrease in the Pittsburgh Sleep Quality Index (PSQI) score, between inclusion and 3 months of follow-up.

### Trial design {8}

This multicenter superiority trial is randomized into two parallel groups of the type of “Immediate solution” versus “Delayed solution.” The main interest in that design is to limit the disappointment bias, each group has access to the solution: one immediately on the day of randomization, the second group after 3 months, once the primary objective is assessed. The allocation ratio between the two groups is 1:1.

## Methods: Participants, interventions, and outcomes

### Study setting {9}

The study takes place in France in seven pain centers of academic hospitals and one private neurology practitioner. The list of participating centers is accessible on ClinicalTrials.gov (NCT05058092).

### Eligibility criteria {10}

To be included, patients must meet the following inclusion criteria:

- Age ≥ 18
- Diagnostic of fibromyalgia according to the American College of Rheumatology criteria 2016 [27]
- Fibromyalgia Impact Questionnaire ≥ 39 (moderate and higher forms) on the inclusion day (D0)
- Wrist size compatible with the device
- Having access to a smartphone running under Android 8 or iOS 12 or later
- Accepting the Fibrepik app on smartphone and counting of their steps by their smartphone
- Accepting the installation of the Google Fit app for patients whose smartphone runs on Android (necessary for counting steps)
- Being affiliated to the French social security system or beneficiary of such a system.

If patients have one of the following exclusion criteria, they cannot be included:

- Characterized depressive episode according to the French version of the Diagnostic and Statistical Manual of Mental Disorders, Fifth Edition (DSM-5) [28]
- Substantial change in treatment in the 3 months prior to inclusion and in the months to come: change in analgesic class, introduction of a new treatment, …
- Chronic inflammatory pathology (chronic inflammatory rheumatism, rheumatoid arthritis, psoriatic arthritis, spondyloarthritis, lupus,...)
- Person in civil proceedings
- Having a dermatological pathology on the wrists, such as oozing dermatosis, hyper sweat or an unhealed lesion
- Being tattooed, pierced of having surgical material implanted on both wrists
- Being allergic to metals and/or silicone
- Referred to articles L1121-5 to L1121-8 of the French Public Health Code: pregnant, parturient, or nursing woman; person deprived of liberty by the judicial or administrative decision; person subject to a legal protection measure or unable to express his/her consent; person under psychiatric care
- In a period of exclusion from other interventional research.

### Who will take informed consent? {26a}

Participants are pre-selected on their medical records within the 8 centers renowned for their implication in fibromyalgia expertise and research. If they meet the eligibility criteria, a Clinical Research Assistant (CRA) contacts these patients by phone to inform them of the study and to verify any criteria that were not in the medical record. If the patient is interested to participate, the trial information letter is sent by post or email. The inclusion visit is scheduled at least 8 days after. During the inclusion visit, the physician gives the patient all the information about the study. If the patient agrees to participate, the patient and the physician sign the consent form.

### Additional consent provisions for collection and use of participant data and biological specimens {26b}

N/A. There is neither biological collection nor ancillary study.

## Interventions

### Explanation for the choice of comparators {6b}

The comparator is conventional medical care because there is no reference treatment in France for fibromyalgia. In addition, following guidelines in chronic nociplastic pain disorder, and specifically in fibromyalgia, we do not plan to propose its wristband alone. Indeed, the coaching allows to transmit knowledge (mechanism of action, expected effects, technical handling) in order to make the patient autonomous in the identification and the implementation of an adequate use of the wristband, i.e., that is, appropriate to them according to the daily evolution of their symptoms. This initial therapeutic information, inspired by Therapeutic Patient Education (TPE) methods, is carried out during a one-hour interview on the day the wristband is handed over, and then reinforced by a phone call 7 days later. Follow-up interviews (45 min by phone) are undergone after one and 2 months of use to help patient self-assess their use of the wristband and the impact on their condition. When necessary, posology (the number of sessions, times at which sessions are scheduled, the intervals between sessions) is revised between patient and coach. These interactions with the patient are indissociable key elements of the solution to improve effectiveness. Coaching is a key point of our study, leading to the impossibility to implement “placebo wristband and placebo coaching.”

### Intervention description {11a}

**Fig. 1:**
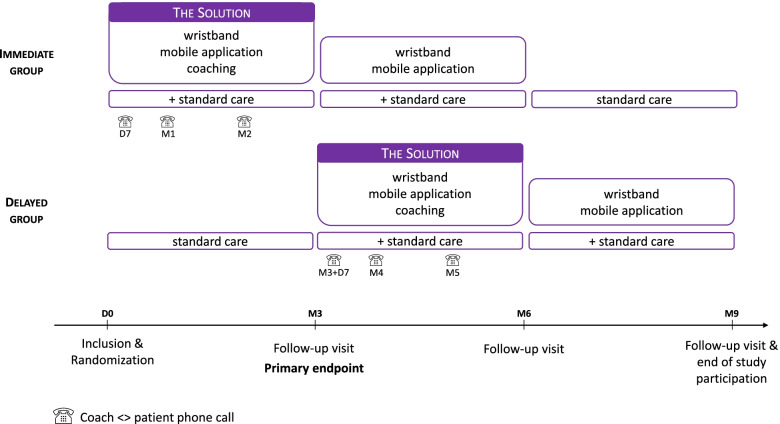
Presentation of the study design

The Figure 1 presents the intervention into the two study groups.

#### Solution description

The solution consists of a wristband, a mobile application, and a coaching program. The wristband is designed to deliver millimeter waves. At the internal part of the band, in contact with the inside of the wrist, two blocks of transmitting antennas and microwave bricks allow to generate the emission of electromagnetic waves of frequency 61.2 GHz with a power density of 13 to 17 mW/cm^2^. A side control button is used to start a session. An indicator light shows that a session is in progress or paused, as well as a possible malfunction. After a session (30 min), the wristband must be recharged by placing it on its charging cradle. Each session is recorded in an internal memory and transferred to the coach via the mobile app.

In this trial, it is recommended that the patient perform at least three sessions per day, including one in the hour before bedtime to facilitate sleep onset and increase sleep quality. Additional sessions may be added if desired.

The Fibrepik application allows the patient to track their sessions and the number of steps taken each day. Through the application, the patient can receive push notifications sent by their coach in case of poor compliance for example. When the patient connects the wristband to the application, usage data is sent to a server, which allows for remote collection of the frequency of use and the distribution of sessions during the day. Access to this data will enrich the understanding of the clinical trial results and are used by the coach, via a secure access, to better adapt their recommendations to each patient during the coaching sessions.

Coaching is key to improve patient adherence to the technology and to increase compliance and effectiveness of the treatment. Coaching is provided by CRA, psychologists, or nurses from each clinical center, specifically trained by a neuropsychologist before the beginning of the inclusions. Four coaching sessions are realized from the delivery of the wristband:

1. At D0 (or M3), upon delivery of the wristband, the patient is trained to use the wristband and the application and is informed about endorphins and their effects in the body. Recommendations for scheduling sessions are made according to the patient’s main symptoms.
2. At D7 (or M3+7 days), a phone call is made to ensure that the patient has no difficulties in using the device, that they have drawn up a routine to use it and to answer questions.
3. At M1 (or M4) and M2 (M5): a phone call, during which an assessment is made of the patient’s usage and impressions of change on their condition. If necessary, the coach provides advice on when and how often to use the wristband.

To standardize the three phone calls, coaches follow dedicated questionnaires that guide the exchange and address the technical aspects, the frequency and habits of use of the patient, the effects felt, ... To evaluate the autonomy of the patient over the following months, coaching does not continue beyond 3 months after patients have started using their wristband.

#### Immediate solution description

The “Immediate solution” group consists of (i) from D0 to M3—3 months with the solution (wristband, Fibrepik App, and coaching) in addition to the standard of care, (ii) from M3 to M6—3 months with the wristband and Fibrepik App in addition to the standard of care, and (iii) from M6 to M9—3 months with the standard of care. These 3 last months with the standard of care is to evaluate the reminiscence effects of the millimeter waves.

#### Delayed solution description

The “Delayed solution” group consists of (i) from D0 to M3—3 months with the standard of care, *ii) from M3 to M6—3 months with the solution add to the standard of care, and (iii) from M6 to M9—3 months with the wristband and Fibrepik App in addition to the standard of care.

To avoid disappointment and lack of investment in the study by the Delayed group, we chose to provide the solution to this group after the collection of the main objective. The fact that these fibromyalgia patients could not benefit from the proposed innovative solution but only continue their usual treatment would have led to many patients refusing to participate or withdrawing consent. Since fibromyalgia is a long-term chronic disease, the 3-month delay for the Delayed solution group to benefit from the solution seems to be a very short period and perfectly acceptable.

### Criteria for discontinuing or modifying allocated interventions {11b}

Patients who could no longer use the wristband because of an adverse effect will stop the treatment. However, they will be followed in the study until the end of their participation. In case of a serious adverse event that no longer allows the patient to participate in the study, the patient’s participation will be stopped. The reason for any discontinuation of treatment or study will be documented and recorded.

### Strategies to improve adherence to interventions {11c}

The coach has access for each patient to the data of the Fibrepik application: the wristband usage data and step counts. If necessary, during the first 3 months of the wristband use, the coach can send a notification to the patient’s smartphone to remind them, for example, to perform sessions or to reinforce the planned coaching sessions scheduled at 7 days (D7) and then after one and 2 months of starting to use the solution. Sending a notification is also used to remind patients in both groups to complete their home follow-up booklet.

### Relevant concomitant care permitted or prohibited during the trial {11d}

All drug treatments or complementary therapies already initiated before the start of the study may be continued but the patient must not have any substantial modification of treatment (change in analgesic class, introduction of a new treatment) during the trial participation. At the end of each week, the patient reports the treatments and complementary therapies taken in their home follow-up booklet.

### Provisions for post-trial care {30}

At the end of the trial, if the patient wishes to continue to use the wristband, they may keep it in addition to conventional care for people with fibromyalgia.

If a serious adverse effect occurs during the trial, the sponsor's insurance would cover the cost of compensation.

### Outcomes {12}

The primary outcome is to compare between the two randomization groups the percentage of patients who significantly improve their fibromyalgia-specific quality of life between the inclusion visit at D0 and the 3-month visit (M3). The fibromyalgia-specific quality of life is evaluated with the French-validated version of the self-questionnaire Fibromyalgia Impact Questionnaire (FIQ). A decrease in FIQ score of 14% or more is considered clinically meaningful [29].

In secondary outcomes number 1 to 5, we compared between the two groups the evolution from D0 to M3 of criteria related to the symptoms of fibromyalgia patients:

1. Questionnaires: Quality of sleep on the Pittsburg Sleep Quality Index (PSQI); Anxiety and depression on the Hospital and Anxiety Depression scale (HAD); Fatigue on the Multidimensional Fatigue Inventory questionnaire (MFI 20); General quality of life on the EQ-5D-5L; Physical activity on the Global Physical Activity Questionnaire (GPAC)
2. Average pain score over the week on a visual analogic scale (VAS)
3. Class, dose, and the number of analgesic, antidepressant, and sleeping pill intake. Patient-reported dose and number of these treatments in his home follow-up booklet
4. Consumption of care in relation to the symptoms of fibromyalgia reported each week by the patient in his home follow-up booklet: care (procedures, medical consultations, hospitalizations); complementary care (acupuncture, osteopath, naturopath, etc.); psycho-behavioral therapies; complementary treatments (phytotherapy, homeopathy, food supplements)
5. Number of steps measured by the subject’s smartphone and collected through the Fibrepik mobile application.

In secondary outcomes 6 to 9, we evaluated:

6. Impression of the disease change at M3 by the patient on the Patient Global Impression of Change (PGIC) scale and by the caregiver on the Clinician Global Impression of Change (CGIC) scale
7. Solution and wristband objectives: Frequency of use of the wristband on the 6-month period of use it. The frequency of use is an extract of the wristband log file; Usability of the wristband on the modular evaluation of key Components of User Experience (meCUE) questionnaire at M6 for patients of the Immediate solution group and at M9 for patients of the Delayed solution group; Solution satisfaction questionnaire (questionnaire created by the sponsor) at M6 for patients of Immediate solution group and at M9 for patients of Delayed solution group
8. Number, description, and classification (serious/non-serious) of adverse effects
9. Primary outcome and secondary outcomes 1 to 6 evaluated at 6 and 9 months.

### Participant timeline {13}

Each patient participates in the study for a period of 9 months. The patient benefit from a consultation with the physician on the day of inclusion at D0 and then during three follow-up visits at 3 (M3), 6 (M6), and 9 (M9) months (Table 1). During each consultation, the outcomes listed above are collected. Randomization is performed by the coach at D0 and if the patient is in the Immediate solution group, he gives the wristband to the patient and performs the first coaching session. For the Delayed solution group, the delivery of the wristband and the first coaching session are performed at 3 months. For both groups, the coach conducted the three other coaching sessions by telephone after 7 days, 1 and 2 months of wristband’s use. A follow-up booklet is given to the patient at D0. The patient must report on it at the end of each week their consumption of drug and complementary treatments. One week per month, the patient indicates each day the pain score on a VAS. At the end of each month, he indicates the activity carried out during the month as well as the medical and paramedical consultations he has received.

**Table 1:** Study schedule of enrolment, follow-up, and assessments

Name of the visit	Eligibility screen	Enrolment	Follow-up			Phone calls							
Temporality	D0–3W	D0	M3	M6	M9	D7	M1	M2	M3+7D	M4	M5	M7	M8
Time window for the visit	± 2W		[− 1W/+ 2W]			±2D	± 1W		±2D	± 1W			
Location of the visit	By phone	Pain center											
Information	✓	✓											
Informed consent		✓											
Clinical examination^a^		✓	✓	✓	✓								
Urine HCG if necessary		✓											
Questionnaires^b^		✓	✓	✓	✓								
Randomization		✓											
Collection of adverse events			✓	✓	✓	✓I	✓I	✓I	✓D	✓D	✓D	✓ID	✓ID
Personalized coaching^c^		✓I	✓D			✓I	✓I	✓I	✓D	✓D	✓D		
Reminder to complete the follow-up booklet							✓D	✓D		✓I	✓I	✓ ID	✓ ID

*M* month, *W* week, *D* day

^a^Clinical examination: height, weight, PAS, PAD, and pain on VAS

^b^Questionnaires: FIQ/PSQI/HAD/IMF20/EQ 5D-5L/GPAC/PGIC and CGIC (not at D0)

^c^Personalized coaching: in each box, it is specified for which group(s) it is realized. *I* Immediate solution, *D* Delayed solution

### Sample size {14}

Considering an alpha risk of 5% and a beta risk of 10%, and assumptions of a rate of patients with a clinically relevant decrease in FIQ of 50% in the Immediate solution group, versus 25% in the Delayed solution group, the number of subjects needed for a comparison of proportions is 77 per group (154 in total). Considering a 10% risk of loss to follow-up, the number of 85 patients per group will be retained (170 patients in total). The clinically relevant decline retained is 14% on the FIQ scale [29].

### Recruitment {15}

Each of the eight centers, renowned for their implication in fibromyalgia expertise and research, estimated its potential for inclusion based on their active patient file already followed within their center and on the availability of human resources (coaches and CRA). Thus, we reach an estimated recruitment of 230 patients over 12 months, which allows us to guarantee the inclusion of 170 patients in 12 months. The main limitation to patients’ recruitment is not the identification of eligible patients but the availability of coaches to provide coaching sessions at D0, D7, M1, and M2.

## Assignment of interventions: allocation

### Sequence generation {16a}

The randomization list includes random blocks of two different sizes. The block sizes were chosen to be small, to prevent group imbalance in the case of an early interruption of the study. The randomization is stratified by center and on the two levels of fibromyalgia severity assessed by the score on the FIQ questionnaire at inclusion day: moderate form (i.e 39 ≤ FIQ score < 59) or severe form (FIQ score ≥ 59) [29].

### Concealment mechanism {16b}

The randomization is performed using the Interactive Web-Response System module of Clininfo (Clininfo SAS – Lyon France - http://www.clininfo.fr/) which is an online central randomization service directly integrated into the eCRF. This platform is available 24h/24 and is compliant with the FDA 21 CFR part 11. Access to this randomization tool is only possible with a coach profile on the eCRF. For each coach of the study, a profile is created, and a login and password are provided by the Remdee Labs datamanager.

The following patient information will be uploaded to Clininfo eCRF before accessing the randomization button: first name and surname initials to create the patient; inclusion and exclusion criteria; Fibromyalgia Impact Questionnaire completed by the patient before inclusion. The score of this questionnaire is automatically calculated on the eCRF and used as a stratifying variable for the randomization. Then the coach clicks on the randomize button and the patient's group is displayed on the computer screen.

### Implementation {16c}

The randomization list has been generated before the start of the trial by a biostatistician of the Grenoble Alps University Hospital data engineering unit. Patients are included in the study by the investigating physicians during a medical consultation. Following this consultation, patients are referred to the coach who enters the data listed above into the eCRF (if the physician has not done this) and then performs the randomization.

## Assignment of interventions: Blinding

### Who will be blinded {17a}

Physicians are blinded to the randomization group while the coach and the patient know it. To guarantee the blindness of the physician, only the coach may randomize the patient and physicians can’t see the patient group on the eCRF. At each follow-up visit, the patient meets the coach first and then the physician. At the M3 visit, the coach reviews the first 3 months of use of the solution for the Immediate group and conducts the first coaching session for the Delayed group patient. So, this ensures patients from both groups have the wristband with them for consultation with the physician. The coach reminds the patient to keep quiet about his group during the consultation with the physician.

### Procedure for unblinding if needed {17b}

Not applicable. This study is on simple blinding: physicians do not know the group while coaches and patients are unblinded.

## Data collection and management

### Plans for assessment and collection of outcomes {18a}

All questionnaires except the GPAC and CGIC are self-administered questionnaires completed by the patient either on paper or directly on a patient secure access to the eCRF. Instructions are given to the patient to complete each questionnaire.

The Fibromyalgia Impact Questionnaire (FIQ) is composed of 10 questions. The first question contains 11 items related to the ability to perform large muscle tasks - each question is rated on a 4-point Likert-type scale. Items 2 and 3 ask the patient to mark the number of days they felt well and the number of days they were unable to work (including housework) because of fibromyalgia symptoms. Items 4 through 10 are horizontal linear scales on which the patient rates work difficulty, pain, fatigue, morning tiredness, stiffness, anxiety, and depression. The French version has been validated by Perrot et al. [30].

The Pittsburg Sleep Quality Index questionnaire is a 19-item, self-rated questionnaire designed to measure sleep quality and disturbance over the past month in clinical populations. The 19 items are grouped into 7 components, including (1) sleep duration, (2) sleep disturbance, (3) sleep latency, (4) daytime dysfunction due to sleepiness, (5) sleep efficiency, (6) overall sleep quality, and (7) sleep medication use. Each of the sleep components yields a score ranging from 0 to 3, with 3 indicating the greatest dysfunction. The sleep component scores are summed to yield a total score ranging from 0 to 21 with the higher total score (referred to as the global score) indicating worse sleep quality. In distinguishing between good and poor sleepers, a global PSQI score > 5 yields a sensitivity of 89.6% and a specificity of 86.5% [31].

The score of the VAS is a real number between 0 and 10. The higher score (10) means the worse pain.

The HAD is an instrument for detecting anxiety and depressive disorders. It was validated and adapted in French [32]. This scale has 14 items rated from 0 to 3 and covers two dimensions. Seven questions are related to the anxiety dimension and seven are related to the depressive dimension, yielding two scores: A (anxiety) and D (depression). The maximum score for each dimension is 21. A score of 11 or higher indicates the probable presence of the disorder.

The Multidimentional Fatigue Inventory is a 20-item scale designed to evaluate five dimensions of fatigue: general fatigue, physical fatigue, reduced motivation, reduced activity, and mental fatigue. The score of each dimension is between 4 and 20. Higher score correspond with more acute levels of fatigue [33].

The EQ-5D-5L is a standardized instrument developed by the Euroquol Group as a measure of health-related quality of life. The EQ-5D-5L contains 5 questions (5Q) on mobility, self-care, usual activities, pain/discomfort, and anxiety/depression with responses rated in 5 levels (5L) and a visual analog scale between 0 and 100. Responses of the 5 questions result to an index [34].

The Global Physical Activity Questionnaire (GPAC) is a questionnaire for the adult population constituted of 16 questions that measure physical and sports activities during a typical week [35]. It aims to collect information on the practice of physical activities (frequency, duration, and intensity of activities) and on sedentary behaviors. The questionnaire covers the following topics: activities at work, during transport, and leisure activities. The results provide a score to classify individuals according to 3 levels of physical activity: low, medium, and high.

The Patient Global Impression of Change and the Clinician Global Impression of Change scales are a 7-point scale depicting a patient's rating of overall improvement [36].

The modular evaluation of key Components of User Experience questionnaire (meCUE) includes 30 items divided into four independent modules: product perception, emotions, consequence of use, and global evaluation. For modules 1 to 3, the user indicates their level of agreement with statements on a 7-point Likert scale, from “strongly disagree” to “strongly agree.” The last question of the meCUE (module 4) asks the user to give an overall evaluation of the product on a scale from − 5 (bad) to + 5 (good) [37].

The solution satisfaction questionnaire (questionnaire created by the sponsor) is a specific questionnaire to the solution including three parts in order to evaluate the patient's satisfaction with the wristband, the mobile application, and the coaching program. A global score between 0 and 100 is given for each part and qualitative questions allow us to better understand this global score.

### Plans to promote participant retention and complete follow-up {18b}

The primary endpoint is collected by a self-questionnaire at D0 and M3. This questionnaire can be completed by the patient directly on the eCRF. Thus, if the patient is unable to attend the follow-up consultation, it will be easy to collect this endpoint. Because of the impact of their symptoms on their quality of life, fibromyalgia patients are generally waiting for a consultation with a physician at the pain center. Thus, we expect very few patients lost to follow-up.

### Data management {19}

Data are collected in an eCRF by the investigating team of each center. The patient can also have a secure access to this eCRF to complete his follow-up booklet at the end of each week and the follow-up questionnaire. This eCRF tool has a data control system to identify data consistency errors, missing data or outliers as soon as they are entered. The data manager also has specific access to request corrections from the investigating team in case of erroneous or missing data. The eCRF includes an audit trail that allows to know for each variable the identity of the person who entered or modified a data as well as the date and time of these actions.

### Confidentiality {27}

In the eCRF, patients are identified by a code composed of the first initial of the surname and first name and the inclusion number in each center. A nominal list linking the patient’s identity to this eCRF identifier is kept in the center by the principal investigator. The data from the wristbands are also non-nominal. When the Fibrepik mobile application is installed, a unique identifier is given to the patient. It is composed of the center number and the patient's inclusion number in the center.

### Plans for collection, laboratory evaluation, and storage of biological specimens for genetic or molecular analysis in this trial/future use {33}

Not applicable. They are no biological specimen collected in this trial.

## Statistical methods

### Statistical methods for primary and secondary outcomes {20a}

Descriptive analyses will be expressed by means − standard deviations and medians − interquartiles for quantitative data, as well as numbers and percentages for qualitative data.

The analysis of the primary endpoint will be performed using a Fisher exact test. For secondary endpoints at M3, all endpoints evaluating a change in score between D0 and M3 will be analyzed via bivariate linear regressions, considering the randomization group and the value of the score at D0. Qualitative criteria will be analyzed using a Fisher test. The secondary endpoint 8 is descriptive and will follow indications for descriptive analysis. For secondary endpoint 9, since analysis considers several measurement points with different states, mixed regressions (linear or logistic depending on the type of variable) will be implemented. Data at M6 from the delayed intervention group will be analyzed for comparison at M3 via a McNemars test (test of proportions for paired data).

These analyses are planned in two stages: (i) all criteria evaluated at 3 months will be performed when all patients have made their follow-up visit at M3 and (ii) all criteria evaluated at 6 and 9 months will be analyzed when the follow-ups at M9 of all patients are completed.

The results will be presented in accordance with the recommendations of the CONSORT Statement. These analyses will be implemented after a consistency check and a freeze of the database, in accordance with the good practices of the Grenoble Alps University Hospital. The software used will be Stata 15 or a more recent version (StataCorp LLC).

### Interim analyses {21b}

Not applicable. There are no interim analyses planned.

### Methods for additional analyses (e.g., subgroup analyses) {20b}

A subgroup analysis according to stratification criteria “center” and “severity of disease” will be performed.

### Methods in analysis to handle protocol non-adherence and any statistical methods to handle missing data {20c}

All analyses will be performed in Intention-To-Treat. In addition to this analysis, a per-protocol analysis will be performed by keeping only patients who have been compliant with the treatment. We consider that a patient is compliant if during the first 3 months of solution use, we observe at least 80% of days with two or more sessions per day. This number of sessions is an extract of the wristband log file.

Missing data within the FIQ questionnaire will be accounted for following the recommendations of the scale authors [38]. A sensitivity analysis of the primary endpoint will be performed by imputing missing data not managed via the FIQ use recommendations via multiple imputation if these missing data represent between 5 and 20% of the total data.

### Plans to give access to the full protocol, participant-level data and statistical code {31c}

The protocol is available on request from the sponsor. At this time, there are no plans to provide access to the participant-level data and statistical code.

## Oversight and monitoring

### Composition of the coordinating center and trial steering committee {5d}

The coordination of the trial is carried out within the sponsor’s medical team. It is composed of the company’s scientific manager, a neuropsychologist, a clinical trial coordinator, and a data manager. The neuropsychologist provides initial training of coaches and, if needed, provides guidance to coaches for their first coaching sessions and throughout the study. The trial coordinator ensures that the trial runs smoothly from both a clinical and a material point of view. The data manager ensures the quality of the data entered in the eCRF to limit the number of missing or aberrant data. This coordination team meets on a weekly basis.

In this study, there is no steering committee.

### Composition of the data monitoring committee, its role, and reporting structure {21a}

There are no data monitoring committees because the primary outcome is a self-questionnaire completed by the patient, and moreover, this study is considered by the French law like a low or negligible risk study. A data monitoring is subcontracted to the Research and Innovation Department of Grenoble Alps University Hospital. A monitor Clinical Research Assistant visit each center at least once a year to ensure that: informed consent is signed from each patient, the clinical center respects the clinical protocol, the data are complete and accurate, and the source data are present in the center.

### Adverse event reporting and harms {22}

This clinical study is considered by the French law like a low or negligible risk study. So, the vigilance of the clinical trial is identical to that of medical care. However, any adverse event is collected immediately in the eCRF and the sponsor is informed by an email alert from the eCRF. If it is an adverse effect, an analysis of the internal data of the wristband is performed to best describe this event. In case of serious adverse effects, a declaration to the health authority would be made by the sponsor.

### Frequency and plans for auditing trial conduct {23}

An audit conducted at the request of the sponsor or an inspection conducted by the health authorities may be carried out at any time by persons who are independent of those responsible for the research. The objective is to ensure the quality of the research, the validity of its results, and compliance with the law and regulations in force. The auditors/inspectors must have direct access to the source and medical data and to any useful document related to the conduct of the clinical study. The confidentiality of the data and the anonymity of the subjects will be respected. Investigators agree to comply with the requirements of the sponsor and the competent authority with respect to an audit or inspection of the research.

### Plans for communicating important protocol amendments to relevant parties (e.g., trial participants, ethical committees) {25}

In case of major modifications of the protocol, an authorization will be requested from the ethics committee. Once approved, the new protocol version will be distributed to the research teams of each center and to the structure responsible for the data monitoring.

### Dissemination plans {31a}

Trial results will be presented to the staff of the different centers involved in the study. They will also be published in a peer-reviewed journal as well as in national and international conferences. A summary of the results can be given to patients who participated on their request.

## Discussion

The aim of this multicenter, randomized, and controlled study is to demonstrate the effectiveness of the integrative approach based on endorphins stimulation and a coaching program for patient suffering from fibromyalgia. If the effectiveness of the solution is demonstrated, we will be able to respond to the demand of fibromyalgia patients for access to an effective non-medicinal treatment to improve their quality of life, and confirm the key recommendation for nociplastic pain, emitted by the EULAR: drug-free approach as first intention and multidisciplinary care [39]. The randomization is of type immediate intervention vs delayed intervention. The Delayed group is the control group that receives the solution after the evaluation of the primary endpoint at 3 months. During these first 3 months, this group receives standard care, so we compare the solution to standard care. The purpose of this methodology is to limit deception bias and facilitate inclusion.

It is recommended that patients use the wristband for a minimum of 3 sessions per day. One of the strengths of this protocol is the follow-up in real-time and for each patient of the realization of the sessions thanks to the extraction of the data from the wristband to the smartphone application. This allows the patient to know at any time of the day the number of sessions performed. This data also allows coaches to remotely monitor their patients’ compliance. In case of poor compliance, the coach can recommend to the patient to increase the frequency of use during a coaching session or by sending a notification to the patient's smartphone. This objectivized compliance with real data will allow us to carry out our per-protocol analysis with patients having completed at least 80% of days with two or more sessions per day.

One of the weaknesses of this protocol is that it is not double-blind, as most of the non-medication and multi-disciplinary clinical trial, as it is impossible to use a placebo arm. However, the primary endpoint and most of the secondary endpoints are patient self-reports.

In addition, patients are encouraged to complete their questionnaires and booklet directly on the eCRF at home, which makes these data independent of any clinician or coach judgment.

## Trial status

At the time of submission, the version of the protocol is V3.0 filed on March 11, 2022. Inclusions started on November 15, 2021, and ended on April 1, 2022. The 9-month follow-up per patient is underway. We had initially planned a 12-month period to include all 170 patients. When the start of the study was announced, the patients followed in each of the 8 centers contacted their physicians to express their wish to participate in this clinical study, and thus, the inclusions were carried out extremely quickly for a clinical trial of this size.

## Supplementary Information

**Additional file 1.** It is the translation of the French ethics approval of the study.

## Data Availability

The final database will be kept by the subcontractor who performs the statistical analyses for 15 years. A copy will be given to the sponsor who will archive it for 15 years once. No data can be reused without the agreement of the sponsor and without compliance with French regulations.

## Abbreviations

ACR: American College of Rheumatology
CGIC: Clinician Global Impression of Change
CRA: Clinical Research Assistant
DSM: Diagnostic and Statistical Manual of mental disorder
eCRF: Electronic case report form
EQ-5D-5L: Euroquol - 5 dimensions – 5 levels
EULAR: European League Against Rheumatism
FIQ: Fibromyalgia Impact Questionnaire
FM: Fibromyalgia
GPAC: Global Physical Activity
HAD: Hospital and Anxiety Depression
meCUE: Modular evaluation of key Components of User Experience
MFI: Multidimensional Fatigue Inventory
MMW: Millimeter waves
PGIC: Patient Global Impression of Change
PSQI: Pittsburg Sleep Quality Index
QoL: Quality of life
TPE: Therapeutic Patient Education
VAS: Visual analogic scale
