# Supplementary material for: A drug free solution for improving the quality of life of fibromyalgia patients (Fibrepik): study protocol of a multicenter, randomized, controlled effectiveness trial
Source: Trials. 2022 Sep 5;23:740. doi: 10.1186/s13063-022-06693-z (PMC9442919; doi:10.1186/s13063-022-06693-z)
Supplement: Supplementary file 1 — Additional file 1. It is the translation of the French ethics approval of the study. [file 13063_2022_6693_MOESM1_ESM.zip › Appendix_FAEC_Fibrepik_EnglishR1.pdf]

DeepL

Subscribe to DeepL Pro to translate larger documents.
Visit www.DeepL.com/pro for more information.

APPENDIX TO THE REQUEST FOR PAC ADVICE FORM

G. General Information about the trial

Primary Objective:

Compare between the two groups the percentage of patients who improve their fibromyalgia-specific quality of life significantly between the inclusion visit at D0 and the 3-month visit (M3). A decrease in FIQ score $\geq$ 14% is considered clinically meaningful (Bennett et al., 2009).

Secondary Objectives:

Secondary objective 1: To compare between the two groups the evolution of sleep quality between D0 and M3.

Secondary objective 2: to compare between the two groups the evolution of pain between D0 and M3

Secondary objective 3: To compare between the two groups the evolution of anxiety and depression between D0 and M3.

Secondary objective 4: to compare between the two groups the evolution of fatigue between D0 and M3.

Secondary objective 5: To compare between the two groups the evolution of analgesic, antidepressant and sleeping pill intake between D0 and M3.

Secondary objective 6: To compare between the two groups the consumption of care related to fibromyalgia between D0 and M3.

Secondary objective 7: To compare between the two groups the change in general quality of life between D0 and M3.

Secondary objective 8: To compare between the two groups the evolution of physical activity between D0 and M3.

Secondary Objective 9: To describe for each of the two groups the impression on the change of the disease by the patient and by the caregiver at M3.

Secondary Objective 10: To characterize the usage data of the Remedee Endorphin Band and the usability and satisfaction of the solution for patients in both groups after six months of solution use.

Secondary Objective 11: To evaluate the adverse effects of the millimeter-wave bracelet for patients in both groups.

Secondary Objective 12: Descriptive analysis of all previous endpoints at M6 and M9.

Main inclusion criteria:

Patients meeting the following criteria will be included in the study:

- major,
- clinical diagnosis of fibromyalgia according to the American College of Rheumatology criteria (Wolfe et al., 2016),
- FIQ score ≥ 39 (moderate and higher forms) on the day of inclusion,
- with a smartphone running Android 8 and iOS 11 or higher,
- accepting the installation of the Fibrepik application on the smartphone,
- accepting the collection of the number of steps measured by the smartphone,
- accepting the installation of the Google Fit application for patients whose smartphone runs on Android (necessary for the collection of the number of steps),
- wrist size compatible with the size M or L of the bracelet,
- affiliated with the social security system or beneficiary of such a system,
- who have signed a consent to participate

Main criteria for non-inclusion:

Subjects with the criteria listed below will not be eligible for inclusion:

- with severe depression,
- substantial change in treatment in the three months prior to inclusion and in the months to come: change of analgesic class, introduction of a new drug treatment.
- with a chronic inflammatory pathology (chronic inflammatory rheumatism, rheumatoid arthritis, psoriatic arthritis, spondyloarthritis, lupus,...),
- person in the course of civil proceedings,
- having a dermatological pathology on the wrists, such as oozing dermatosis, hyper sweat or an unhealed lesion,
- with a surgical implant, tattoo or piercing on one of the wrists,
- allergic to metals and/or silicone,
- referred to in articles L1121-5 to L1121-8 of the Public Health Code (CSP),
- during a period of exclusion from other interventional research

Primary endpoint(s)

The endpoint will be the FIQ score at D0 and M3.

I. INVESTIGATORS AND RESEARCH SITES

I.2 Other investigators :

N°	CENTER<br>FULL NAME AND ADDRESS / SERVICE	LIST OF INVESTIGATORS<br>(PLEASE INDICATE NAME AND SURNAME)	SPPN
1	Grenoble Alpes<br>University Hospital<br>CETD<br>38019 Grenoble cedex	Coordinating Investigator<br>Caroline MAINDET	10003947586

2	Valencian University Hospital CETD Jean Bernard Hospital Av Desandrouin CS50479 - 59322 Valenciennes cedex	Principal Investigator Antoine LEMAIRE	10004608575
3	Foch Hospital Department of Anesthesia 92150 Suresnes	Principal Investigator Mireille MICHEL-CHERQUI	10000982685
4	Lariboisière Hospital Department of Pain Medicine, Palliative Medicine 2 rue Ambroise Paré, 75475 Paris cedex 10	Principal investigator Alain SERRIE	10000267004
5	Rouen University Hospital CETD 1 rue Germont - 76031 ROUEN CEDEX	Principal Investigator Rodrigue DELEENS	10003750576
6	Private practice of neurology 31 rue Boiron 69440 MORNANT	Principal Investigator Alberta LORENZI-PERNOT	10003072062
7	CHU Montpellier Psychosomatic Pain Department Functional disease Hôpital saint Eloi - 34295 Montpellier cedex 5	Principal Investigator Patrick GINIES	10003216446
8	Medipôle mutualist hospital 158 rue Léon Blum 69100 Villeurbanne	Principal Investigator Mario BARMAKI	10003125233
