# Supplementary material for: A drug free solution for improving the quality of life of fibromyalgia patients (Fibrepik): study protocol of a multicenter, randomized, controlled effectiveness trial
Source: Trials. 2022 Sep 5;23:740. doi: 10.1186/s13063-022-06693-z (PMC9442919; doi:10.1186/s13063-022-06693-z)
Supplement: Supplementary file 1 — Additional file 1. It is the translation of the French ethics approval of the study. [file 13063_2022_6693_MOESM1_ESM.zip › Appendix_FAEC_Fibrepik_FrenchR1.pdf]

ANNEXE AU FORMULAIRE DE DEMANDE D'AVIS AU CPP

G. Informations Générales sur l'essai

Objectif principal :

Comparer entre les deux groupes le pourcentage de patient qui améliore leur qualité de vie spécifique à la fibromyalgie de manière significative entre la visite d'inclusion à J0 et la visite à 3 mois (M3). Une diminution de score FIQ ≥ 14% est considérée comme cliniquement significative (Bennett et al., 2009).

Objectifs secondaires :

Objectif secondaire 1 : comparer entre les deux groupes l'évolution de la qualité du sommeil entre J0 et M3.

Objectif secondaire 2 : comparer entre les deux groupes l'évolution de la douleur entre J0 et M3

Objectif secondaire 3 : comparer entre les deux groupes l'évolution de l'anxiété et de la dépression entre J0 et M3.

Objectif secondaire 4 : comparer entre les deux groupes l'évolution de la fatigue entre J0 et M3.

Objectif secondaire 5 : comparer entre les deux groupes l'évolution de la prise d'antalgique, d'antidépresseur et de somnifère entre J0 et M3.

Objectif secondaire 6 : comparer entre les deux groupes la consommation de soins en lien avec la fibromyalgie entre J0 et M3.

Objectif secondaire 7 : comparer entre les deux groupes l'évolution de la qualité de vie générale entre J0 et M3.

Objectif secondaire 8 : comparer entre les deux groupes l'évolution de l'activité physique entre J0 et M3.

Objectif secondaire 9 : décrire pour chacun des deux groupes l'impression sur le changement de la maladie par le patient et par le soignant à M3.

Objectif secondaire 10 : caractériser les données d'usage du bracelet Remedee Endorphin Band ainsi que l'aptitude à l'utilisation et la satisfaction de la solution pour les patients des deux groupes après six mois d'utilisation de la solution.

Objectif secondaire 11 : évaluer les effets indésirables du bracelet à ondes millimétriques pour les patients des deux groupes.

Objectif secondaire 12 : analyse descriptive de l'ensemble des critères de jugement précédents à M6 et à M9.

Principaux critères d'inclusion :

Seront inclus dans l'étude, les patients répondants aux critères suivants :

- majeurs,
- diagnostic clinique de fibromyalgie selon les critères du collège Américain de Rhumatologie (Wolfe et al., 2016),
- score au FIQ ≥ 39 (formes modérées et supérieures) le jour de l'inclusion,
- disposant d'un smartphone qui fonctionne avec les versions Android 8 et iOS 11 ou plus récentes,
- acceptant l'installation sur le smartphone de l'application Fibrepik,
- acceptant le recueil du nombre de pas mesuré par le smartphone,
- acceptant l'installation de l'application Google Fit pour les patients dont le smartphone fonctionne sous Android (nécessaire pour le recueil du nombre de pas),
- tour de poignet compatible avec le gabarit taille M ou L du bracelet,
- affiliés à la sécurité sociale ou bénéficiaire d'un tel régime,
- ayant signé un consentement de participation

Principaux critères de non inclusion :

Les sujets qui présentent les critères listés ci-dessous ne pourront pas être inclus :
- présentant une dépression sévère,
- modification substantielle de traitement dans les trois mois précédents l'inclusion et les mois à venir : changement de classe d'antalgique, introduction d'un nouveau traitement médicamenteux.
- présentant une pathologie inflammatoire chronique (rhumatisme inflammatoire chronique, polyarthrite rhumatoïde, rhumatismes psoriasiques, spondyloarthrite, lupus,…),
- personne en cours de procédure civile,
- présentant au niveau des poignets une pathologie dermatologique, type dermatose suintante, hyper sudation ou une lésion non cicatrisée,
- présentant un implant chirurgical, un tatouage ou piercing au niveau d'un des poignets,
- allergiques aux métaux et/ou silicone,
- visés aux articles L1121-5 à L1121-8 du Code de la Santé Publique (CSP),
- en période d'exclusion d'une autre recherche interventionnelle

Critère(s) d'évaluation principal (aux)

Le critère de jugement sera le score FIQ à J0 et à M3.

I. INVESTIGATEURS ET LIEUX DE RECHERCHE

I.2. Autres investigateurs :

N°	CENTRE<br>NOM ET ADRESSE COMPLETE / SERVICE	LISTE DES INVESTIGATEURS<br>(VEUILLEZ INDIQUER LE NOM ET PRENOM)	N°RPPS
1	CHU Grenoble Alpes<br>CETD<br>38019 Grenoble cedex	Investigateur coordonnateur<br>Caroline MAINDET	10003947586

2	CHU Valencienne<br>CETD<br>Hôpital Jean Bernard<br>Av Desandrouin<br>CS50479 – 59322 Valenciennes cedex	Investigateur principal<br>Antoine LEMAIRE	10004608575
3	Hôpital Foch<br>Service d'Anesthésie<br>92150 Suresnes	Investigateur principal<br>Mireille MICHEL-CHERQUI	10000982685
4	Hôpital Lariboisière<br>Service de Médecine de la douleur, Médecine palliative<br>2 rue Ambroise Paré,<br>75475 Paris cedex 10	Investigateur principal<br>Alain SERRIE	10000267004
5	CHU Rouen<br>CETD<br>1 rue Germont – 76031 ROUEN CEDEX	Investigateur principal<br>Rodrigue DELEENS	10003750576
6	Cabinet libéral de neurologie<br>31 rue Boiron<br>69440 MORNANT	Investigateur principal<br>Alberta LORENZI-PERNOT	10003072062
7	CHU Montpellier<br>Département Douleur Psychosomatique Maladie fonctionnelle<br>Hôpital saint Eloi - 34295 Montpellier cedex 5	Investigateur principal<br>Patrick GINIES	10003216446
8	Medipôle hôpital mutualiste<br>158 rue Léon Blum<br>69100 Villeurbanne	Investigateur principal<br>Mario BARMAKI	10003125233
